# Clinical prediction model for acute exacerbation of chronic heart failure: Development and application of a nomogram

**DOI:** 10.1097/MD.0000000000048603

**Published:** 2026-05-22

**Authors:** Da Yang, Shuting Zhai, Jian Ma, Fachao Shi

**Affiliations:** a Department of Cardiology, Maanshan People’s Hospital, Maanshan, Anhui, China.

**Keywords:** acute exacerbation, chronic heart failure, nomogram, prognostic assessment, risk prediction

## Abstract

Acute exacerbation of chronic heart failure (CHF) is a major cause of recurrent hospitalization and mortality in heart failure patients. Accurate prediction of acute exacerbation of CHF risk is crucial for optimizing clinical management and improving patient outcomes. This study aimed to develop and validate a simple and practical nomogram model for predicting the risk of acute exacerbation in patients with CHF. This retrospective cohort study included 220 patients with CHF hospitalized in the Department of Cardiology of our institution between January 2019 and December 2023. The patients were randomly divided into a training cohort (n = 154) and a validation cohort (n = 66) at a 7:3 ratio using a random number table. Independent risk factors were identified through least absolute shrinkage and selection operator regression analysis, and a nomogram prediction model was constructed. The discriminative ability and calibration of the model were evaluated using receiver operating characteristic curves, calibration curves, and the Hosmer–Lemeshow goodness-of-fit test. During the 12-month follow-up period, 108 patients (49.09%) experienced acute exacerbation. least absolute shrinkage and selection operator regression analysis identified age, hypertension, left ventricular ejection fraction, coronary heart disease, and lymphocyte count as independent predictors of acute exacerbation of CHF. The nomogram model constructed based on these factors demonstrated area under the curve of 0.838 (95% confidence interval: 0.775–0.902) in the training cohort and 0.712 (95% confidence interval: 0.581–0.843) in the validation cohort. The calibration curves indicated strong agreement between predicted probabilities and actual observed incidence (Hosmer–Lemeshow test: *P* = .8651). This study successfully developed and validated a nomogram model based on readily available clinical indicators that can effectively predict the risk of acute exacerbation in patients with CHF. The model demonstrates good discriminative ability and calibration, aiding clinicians in individualized risk assessment and precise management.

## 1. Introduction

Chronic heart failure (CHF) is a major global public health issue with persistently high morbidity and mortality rates, and its prevalence continues to rise with the accelerating aging of the population.^[1]^ It is estimated that approximately 64 million people worldwide are affected by heart failure, which has a 5-year mortality rate as high as 50%, exceeding that of many malignancies.^[2]^ In the course of this disease, acute heart failure exacerbation (AHFE) represents a critical turning point that leads to repeated hospitalizations, significantly reduced quality of life, and worsened prognosis.^[3]^ About 30 to 40% of discharged patients are readmitted within 6 months due to acute exacerbations, which not only increases the disease burden for patients but also consumes substantial healthcare resources.^[4]^

Currently, there are several predictive models for the long-term prognosis assessment of CHF, such as the Seattle Heart Failure Model and the Meta-Analysis Global Group in Chronic Heart Failure risk score.^[5,6]^ However, most of these models focus primarily on predicting all-cause mortality, while models specifically designed to predict the risk of acute exacerbation events remain relatively scarce.^[7]^ The pathophysiological mechanisms of acute exacerbation involve hemodynamic deterioration, neurohormonal overactivation, and inflammatory responses, among others. Risk factors for AHFE may differ from those for mortality.^[8]^ Therefore, developing a practical tool dedicated to predicting AHFE risk is of significant clinical importance.

In recent years, the nomogram a visual predictive tool based on multivariate regression models has been widely used in medical prognostic research.^[9]^ It can transform complex statistical models into an intuitive graphical interface, enabling clinicians to quickly integrate multiple predictive indicators and quantitatively estimate the probability of specific events for individual patients.^[10]^ In fields such as oncology,^[11]^ cardiology,^[12]^ and other chronic disease management areas,^[13]^ nomograms have demonstrated good predictive accuracy and clinical utility.

Despite these advantages, nomogram models specifically designed for predicting acute exacerbation risk in patients with CHF are still insufficient. Existing prediction models often have limitations, such as reliance on expensive or nonroutine testing indicators,^[14]^ lack of adequate external validation,^[15]^ or poor calibration.^[16]^ Therefore, constructing a well-validated and effective nomogram prediction model based on routine clinical indicators will help identify high-risk patients and provide a basis for implementing targeted intensive management strategies.

This study aims to identify independent predictive factors closely associated with acute exacerbation in CHF using clinical data, and to develop an individualized risk prediction nomogram model. Through internal and external validation, the model’s discriminative ability, calibration, and clinical utility will be evaluated.

## 2. Materials and methods

### 2.1. Study design and subjects

This study adopted a retrospective cohort design and enrolled patients with CHF who were hospitalized in the Department of Cardiology at Maanshan People’s Hospital between January 2019 and December 2023. The study was approved by the Ethics Committee of the participating hospital, and informed consent was waived due to its retrospective nature. Inclusion criteria were as follows: age ≥ 18 years, diagnosis of CHF meeting the criteria outlined in the 2022 European Society of Cardiology Guidelines for the diagnosis and treatment of heart failure^[17]^, disease duration ≥ 3 months, complete clinical data, complete follow-up data. Exclusion criteria included: acute myocardial infarction or unstable angina, severe valvular heart disease, congenital heart disease, malignant tumors, severe hepatic or renal dysfunction, pregnancy or lactation, life expectancy < 12 months, loss to follow-up.

### 2.2. Data collection

All covariates were extracted from the electronic medical record at the index hospitalization. Unless otherwise specified, values reflect the first available measurements on admission and were obtained using standardized hospital procedures and assays. Demographics: age (years) and sex were abstracted from registration records. Body mass index was calculated as weight (kg) divided by height (m) squared. Smoking history was recorded from admission notes; patients with a documented history of cigarette use were classified as ever-smokers, while those without were classified as never-smokers. Comorbidities: hypertension and diabetes were defined by a documented physician diagnosis and/or ongoing use of corresponding antihypertensive or glucose-lowering medications. Chronic kidney disease was defined by a documented physician diagnosis in the medical history. Coronary heart disease (CHD) was defined as a documented history of myocardial infarction, prior coronary revascularization, or clinically diagnosed coronary artery disease supported by imaging (e.g.,invasive angiography or coronary computed tomography angiography) in the medical record. Clinical status and vital signs: New York Heart Association functional class was assigned per guideline-based clinical assessment at admission. Systolic and diastolic blood pressure were recorded as measured at admission and reported in mm Hg. Laboratory measurements: complete blood count included absolute lymphocyte count (primary immune cell marker used in analyses), neutrophil count, hemoglobin, and platelet count. Serum chemistries included albumin, serum creatinine, blood urea nitrogen, uric acid, alanine aminotransferase, aspartate aminotransferase, fasting plasma glucose, glycated hemoglobin, total cholesterol, triglycerides, low-density lipoprotein cholesterol, high-density lipoprotein cholesterol, and B-type natriuretic peptide. All assays were performed by the hospital central laboratory using standardized methods; differential counts refer to absolute values as reported. Cardiac imaging: left ventricular ejection fraction (LVEF, %) was obtained from transthoracic echocardiography on admission and calculated using the biplane Simpson method when available, per standard practice. Coding for analyses: in the modeling dataset, binary comorbidities (e.g. hypertension, diabetes, chronic kidney disease, CHD), sex, and smoking history were coded as 0 = no and 1 = yes. Continuous variables (e.g., age, LVEF, laboratory indices) were used on their native scales without dichotomization. The dependent variable was the occurrence of acute exacerbation of CHF within 12 months (0 = no,1 = yes).

### 2.3. Definition of primary endpoint events

The primary endpoint was defined as an acute exacerbation of CHF within 12 months, meeting any of the following criteria: unplanned hospitalization due to worsening heart failure symptoms; intravenous administration of diuretics, vasodilators, or inotropic agents in an outpatient or emergency department setting due to worsening heart failure; heart failure-related death. Endpoint events were independently assessed by 2 experienced cardiologists. Any discrepancies were resolved by a third cardiologist. Heart failure phenotype was not prespecified for standardized collection. Echocardiographic examinations were obtained per routine clinical care with variable timing and protocols across centers, and key parameters were incompletely recorded. Accordingly, we did not classify patients into heart failure with reduced ejection fraction, heart failure with mildly reduced ejection fraction, or heart failure with preserved ejection fraction (HFpEF), and we do not report subtype frequencies.

### 2.4. Follow-up methods

Follow-up was conducted through outpatient clinic reviews, telephone follow-ups, and electronic medical record reviews. Assessments were carried out at 1, 3, 6, and 12 months after discharge to document changes in symptoms, rehospitalization events, medication adjustments, and occurrence of endpoint events. The follow-up period concluded on December 31, 2024.

### 2.5. Statistical methods

Statistical analyses were performed using SPSS 26.0 (IBM Corp., Armonk) and R software version 4.3.0 (R Foundation for Statistical Computing, Vienna, Austria). Before analysis, all records were screened for completeness. In accordance with the study inclusion criteria (“complete clinical data” and “complete follow-up data”), patients with missing baseline clinical variables, imaging indices, key laboratory tests, or primary endpoint information were excluded prior to analysis. As a result, for the final analytic cohort (n = 220), all candidate predictors used in model development (age, hypertension, LVEF, CHD, and lymphocyte count) and the primary endpoint were complete. Therefore, model building and validation were performed using complete-case data, and no single or multiple imputation was applied. Random allocation to the training (70%) and validation (30%) cohorts was conducted after implementing these completeness criteria. Continuous variables with normal distribution are presented as mean ± standard deviation, while those with non-normal distribution are expressed as median (interquartile range). Categorical variables are summarized as number (percentage). Group comparisons were made using the *t*-test or Mann–Whitney *U* test for continuous variables, and the chi-square test or Fisher exact test for categorical variables.

We used least absolute shrinkage and selection operator (LASSO) logistic regression to perform variable selection and reduce dimensionality. The penalty parameter *λ* was tuned via 10-fold cross-validation in the training cohort, and the λ within 1 standard error (SE) of the minimum cross-validated error (1-SE rule) was selected to favor a more parsimonious model. Predictors with nonzero coefficients at the selected *λ* were retained. The final predictive model was a multivariable logistic regression fitted in the training cohort using the LASSO-selected predictors. Regression coefficients (*β*) and odds ratios with 95% confidence intervals (CI) were estimated. Linearity for continuous predictors on the logit scale was assessed; given no material deviation, predictors were modeled linearly. Candidate 2-way interactions were screened based on clinical plausibility, and none materially improved model fit by likelihood ratio testing; thus, no interaction terms were retained. A nomogram was constructed from the fitted logistic model with point allocations proportional to the *β* coefficients. The total point score maps to the predicted 12-month risk of acute exacerbation. Internal validation was conducted using bootstrap resampling with 1000 replicates in the training cohort to estimate and correct for potential optimism in discrimination and calibration. Discrimination was summarized by the area under the receiver operating characteristic curve (AUC) with 95% CI (DeLong method) and was also reported separately in the independent validation cohort. Calibration was evaluated by calibration plots (bootstrap-corrected) and the Hosmer–Lemeshow goodness-of-fit test. Clinical utility was examined using decision curve analysis to quantify net benefit across a range of threshold probabilities.

## 3. Results

### 3.1. Patient baseline characteristics

A total of 356 patients with CHF were initially screened during the study period. After excluding 106 patients due to incomplete data and 30 patients lost to follow-up, 220 patients were ultimately included. These were divided into a training set (n = 154) and a validation set (n = 66). The median age of the patients was 56.00 (41.75, 68.00) years; 107 (48.6%) were male and 113 (51.4%) were female. According to New York Heart Association functional classification, there were 15 class I, 80 class II, 104 class III, and 21 class IV patients. The median LVEF was 62.00% (57.75, 65.00). No statistically significant differences were observed in baseline characteristics between the training and validation sets (*P* > .05), indicating that the 2 groups were well-balanced (Table 1).

**Table 1:** Comparison of general clinical data between training set and validation set.

Variables	Total (N = 220)	Test data (N = 66)	Train data (N = 154)	*P* Value
Age, Median (Q1, Q3)	56.00 (41.75, 68.00)	58.00 (46.75, 65.00)	55.00 (36.75, 70.00)	.694
BMI, Median (Q1, Q3)	30.05 (25.89, 33.40)	30.25 (26.13, 33.85)	29.94 (25.80, 32.95)	.555
SBP, Median (Q1, Q3)	124.00 (114.00, 137.00)	123.00 (115.00, 144.75)	124.00 (113.00, 135.00)	.462
DBP, Median (Q1, Q3)	70.00 (63.00, 78.00)	70.50 (64.75, 79.25)	68.50 (61.75, 77.00)	.170
FPG, Median (Q1, Q3)	5.83 (5.33, 6.75)	5.88 (5.38, 6.55)	5.80 (5.33, 6.86)	.606
HbA1c, Median (Q1, Q3)	5.70 (5.30, 6.23)	5.75 (5.40, 6.20)	5.70 (5.30, 6.30)	.493
ALT, Median (Q1, Q3)	21.50 (16.00, 29.00)	21.00 (16.00, 26.00)	22.00 (16.00, 31.25)	.228
AST, Median (Q1, Q3)	22.00 (19.00, 28.00)	22.00 (19.00, 25.25)	23.00 (19.00, 29.00)	.211
Albumin, Median (Q1, Q3)	4.20 (4.00, 4.40)	4.15 (3.90, 4.40)	4.20 (4.00, 4.40)	.480
Creatinine, Median (Q1, Q3)	78.68 (65.42, 93.04)	76.91 (67.18, 90.17)	80.44 (63.65, 97.24)	.751
UA, Median (Q1, Q3)	345.00 (279.60, 410.40)	353.90 (273.60, 400.00)	345.00 (285.50, 416.40)	.910
BUN, Median (Q1, Q3)	5.00 (3.93, 6.43)	4.64 (3.84, 5.71)	5.00 (3.93, 6.78)	.320
TG, Median (Q1, Q3)	1.47 (0.99, 2.11)	1.47 (0.98, 2.08)	1.48 (1.00, 2.14)	.743
TC, Median (Q1, Q3)	4.79 (4.22, 5.69)	4.78 (4.26, 5.79)	4.81 (4.21, 5.59)	.729
HDL, Median (Q1, Q3)	1.21 (1.02, 1.53)	1.21 (1.03, 1.45)	1.21 (1.00, 1.53)	.970
LDL, Median (Q1, Q3)	2.77 (2.28, 3.44)	2.79 (2.30, 3.58)	2.74 (2.28, 3.31)	.685
Lymphocyte, Median (Q1, Q3)	3.50 (2.50, 3.60)	3.50 (2.40, 3.60)	3.50 (2.50, 3.60)	.496
Neutrophils, Median (Q1, Q3)	4.20 (3.30, 5.40)	4.50 (3.40, 5.60)	4.10 (3.18, 5.20)	.344
Hemoglobin, Median (Q1, Q3)	14.10 (13.10, 15.00)	14.10 (13.28, 15.00)	14.10 (13.10, 14.90)	.479
Platelet, Median (Q1, Q3)	246.00 (203.75, 289.00)	253.50 (206.75, 295.25)	242.50 (202.75, 285.50)	.275
LVEF, Median (Q1, Q3)	62.00 (57.75, 65.00)	62.00 (56.75, 65.00)	62.00 (58.00, 65.00)	.405
NYHA III-IV, n (%)	180 (56.30)	54 (56.25)	126 (56.25)	.965
BNP, pg/mL	624 (289–1156)	638 (294–1168)	595 (281–1142)	.421
CHF, n (%)				.863
No	112 (50.9%)	32 (50.0%)	80 (51.3%)	
Yes	108 (49.1%)	32 (50.0%)	76 (48.7%)	
Sex, n (%)				.738
Female	113 (51.4%)	34 (53.1%)	79 (50.6%)	
Male	107 (48.6%)	30 (46.9%)	77 (49.4%)	
Smoke, n (%)				.203
Former	53 (24.1%)	15 (23.4%)	38 (24.4%)	
Never	96 (43.6%)	23 (35.9%)	73 (46.8%)	
Now	71 (32.3%)	26 (40.6%)	45 (28.8%)	
Hypertension, n (%)				.072
No	82 (37.3%)	18 (28.1%)	64 (41.0%)	
Yes	138 (62.7%)	46 (71.9%)	92 (59.0%)	
Diabetes, n (%)				.729
No	144 (65.5%)	43 (67.2%)	101 (64.7%)	
Yes	76 (34.5%)	21 (32.8%)	55 (35.3%)	
CHD, n (%)				.029
No	178 (80.9%)	46 (71.9%)	132 (84.6%)	
Yes	42 (19.1%)	18 (28.1%)	24 (15.4%)	
CKD, n (%)				.073
No	149 (67.7%)	49 (76.6%)	100 (64.1%)	
Yes	71 (32.3%)	15 (23.4%)	56 (35.9%)	

ALT = alanine aminotransferase, AST = aspartate aminotransferase, BMI = body mass index, BNP = B-type natriuretic peptide, BUN = blood urea nitrogen, CHD = coronary heart disease, CHF = chronic heart failure, CKD = chronic kidney disease, DBP = diastolic blood pressure, FPG = fasting plasma glucose, HbA1c = glycated hemoglobin, HDL = high-density lipoprotein, LDL = low-density lipoprotein, LVEF = left ventricular ejection fraction, n/N = number of participants, NYHA = New York Heart Association, SBP = systolic blood pressure, TC = total cholesterol, TG = triglycerides, UA = uric acid.

### 3.2. Follow-up results and endpoint events

The median follow-up time was 12.0 months (range: 12.0–15.2 months). During follow-up, a total of 108 patients (33.75%) experienced acute exacerbation of CHF, including 76 cases (48.7%) in the training set and 32 cases (50.0%) in the validation set. The difference in incidence between the 2 groups was not statistically significant (*P* = .863). The distribution of acute exacerbation events was as follows: 94 cases (87.04%) were hospitalized due to worsened heart failure, 8 cases (7.41%) received intravenous medication in outpatient or emergency settings, and 6 cases (5.56%) resulted in heart failure-related death. The majority of acute exacerbations (68.4%) occurred within the first 6 months of follow-up.

### 3.3. Screening for risk factors

**Figure 1.**
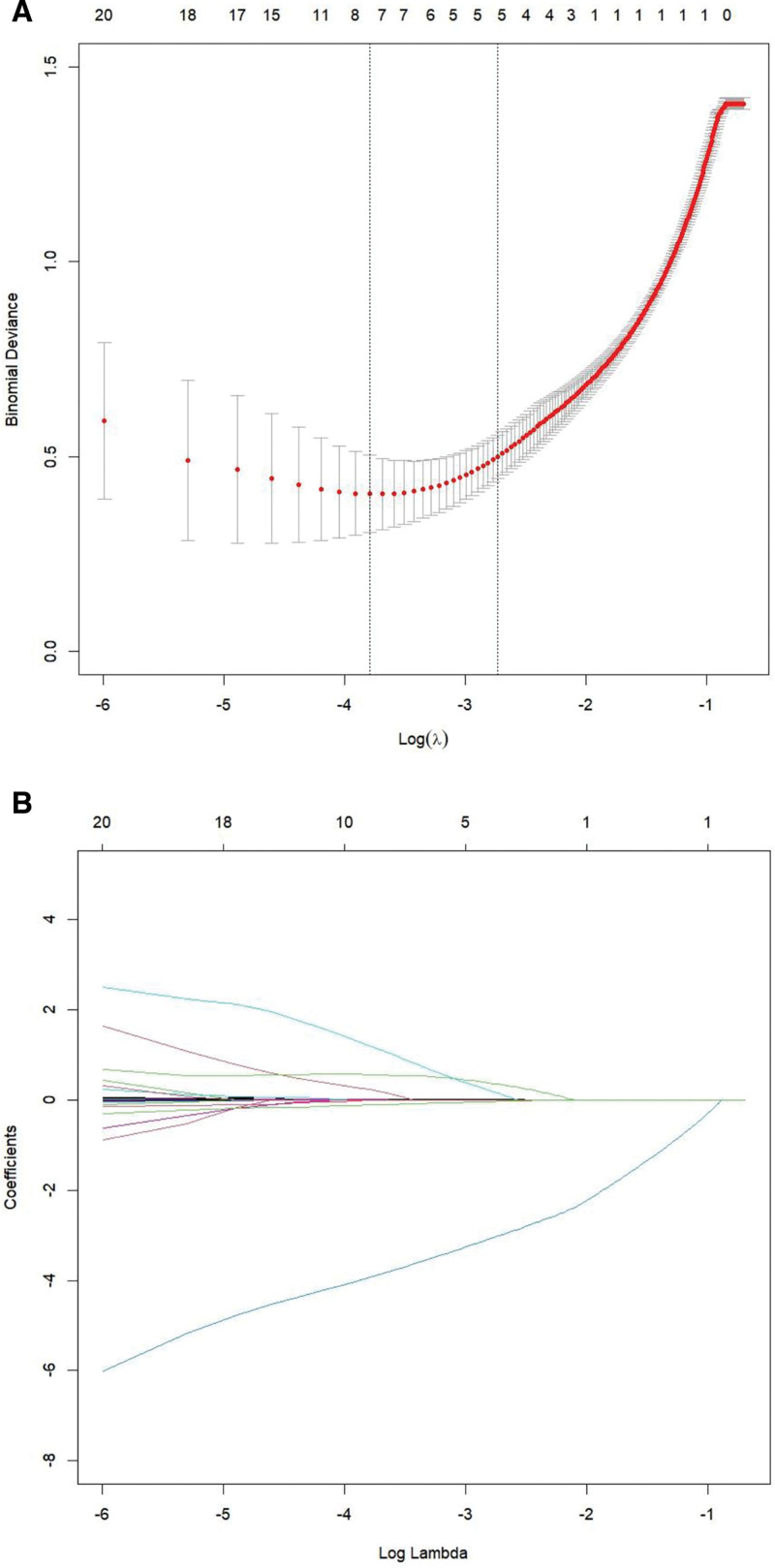
LASSO screening for risk factors of acute exacerbation of CHF. (A) 10-fold cross-validation for tuning parameter λ (binomial deviance); (B) coefficient profiles versus log (λ). CHF = chronic heart failure, LASSO = least absolute shrinkage and selection operator, SE = standard error.

LASSO regression was applied for variable selection and dimensionality reduction. The optimal *λ* value was determined using 10-fold cross-validation and was selected as the value within 1 SE of the minimum mean squared prediction error (Fig. 1). Ultimately, the LASSO regression analysis identified 5 predictors with nonzero coefficients: age, hypertension, LVEF, CHD, and lymphocyte count.

### 3.4. Nomogram model building

**Figure 2.**
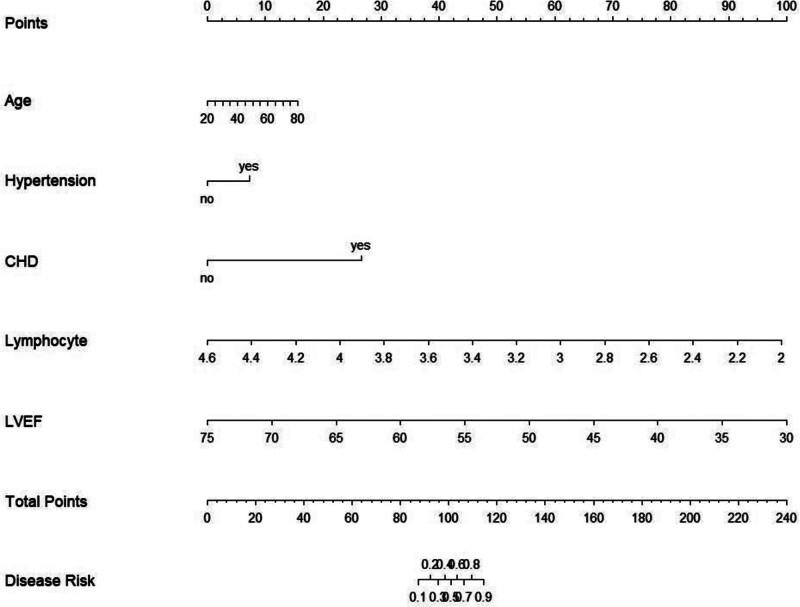
A nomogram model to predict the risk of acute exacerbation of CHF. CHD = chronic heart disease, CHF = chronic heart failure, LVEF = left ventricular ejection fraction.

Based on the results of the LASSO regression analysis, a nomogram model was developed to predict the risk of acute exacerbation in patients with CHF (Fig. 2). In the nomogram, each predictor is assigned a points score. The total points, obtained by summing the individual scores, correspond to the predicted probability of acute exacerbation within 12 months.

### 3.5. Model validation and performance evaluation

**Figure 3.**
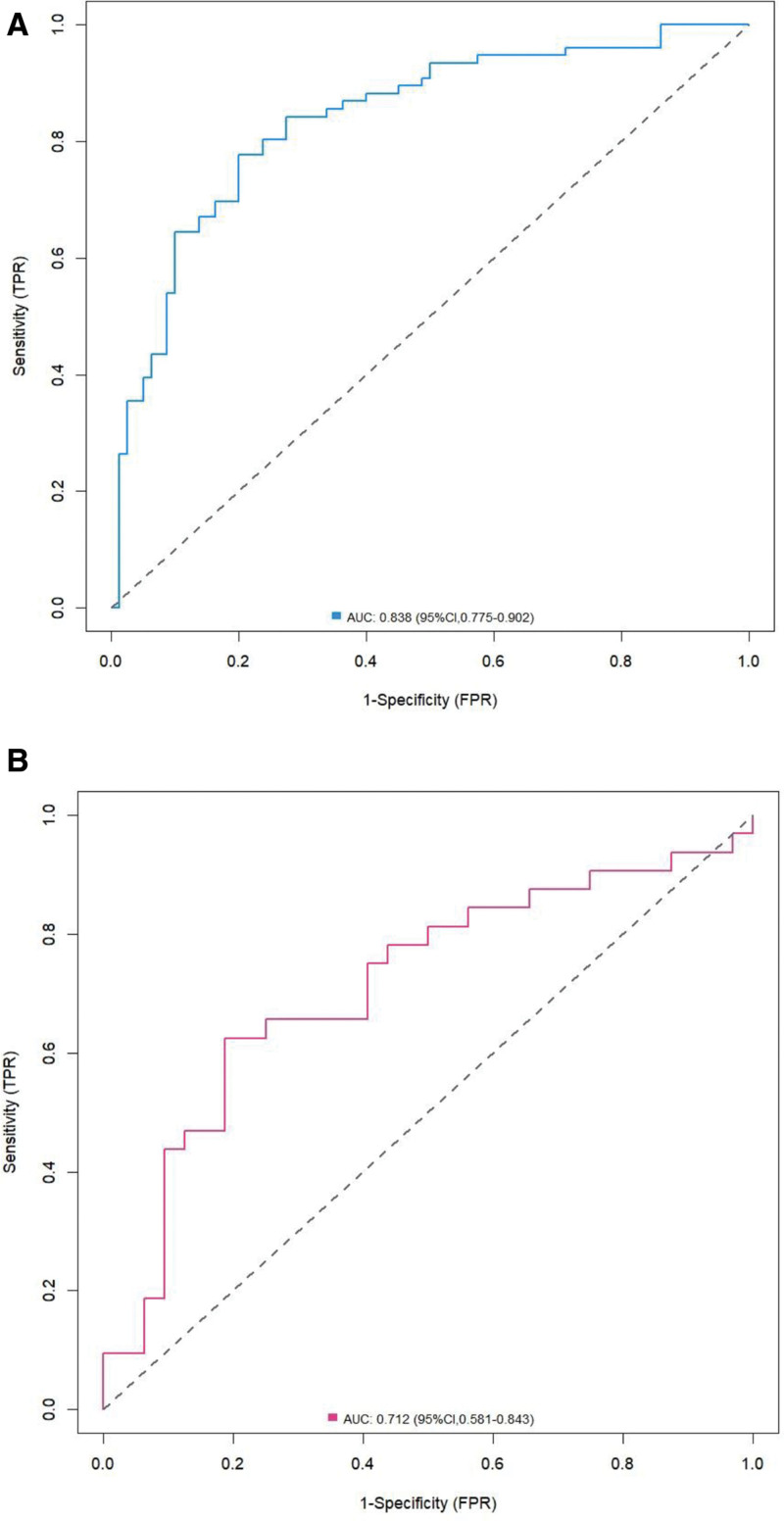
ROC curve analysis of nomogram models for training and validation sets. (A) training set; (B) validation set. AUC = area under the curve, CI = confidence interval, FPR = false positive rate (1-specificity), ROC = receiver operating characteristic, TPR = true positive rate (sensitivity).

**Figure 4.**
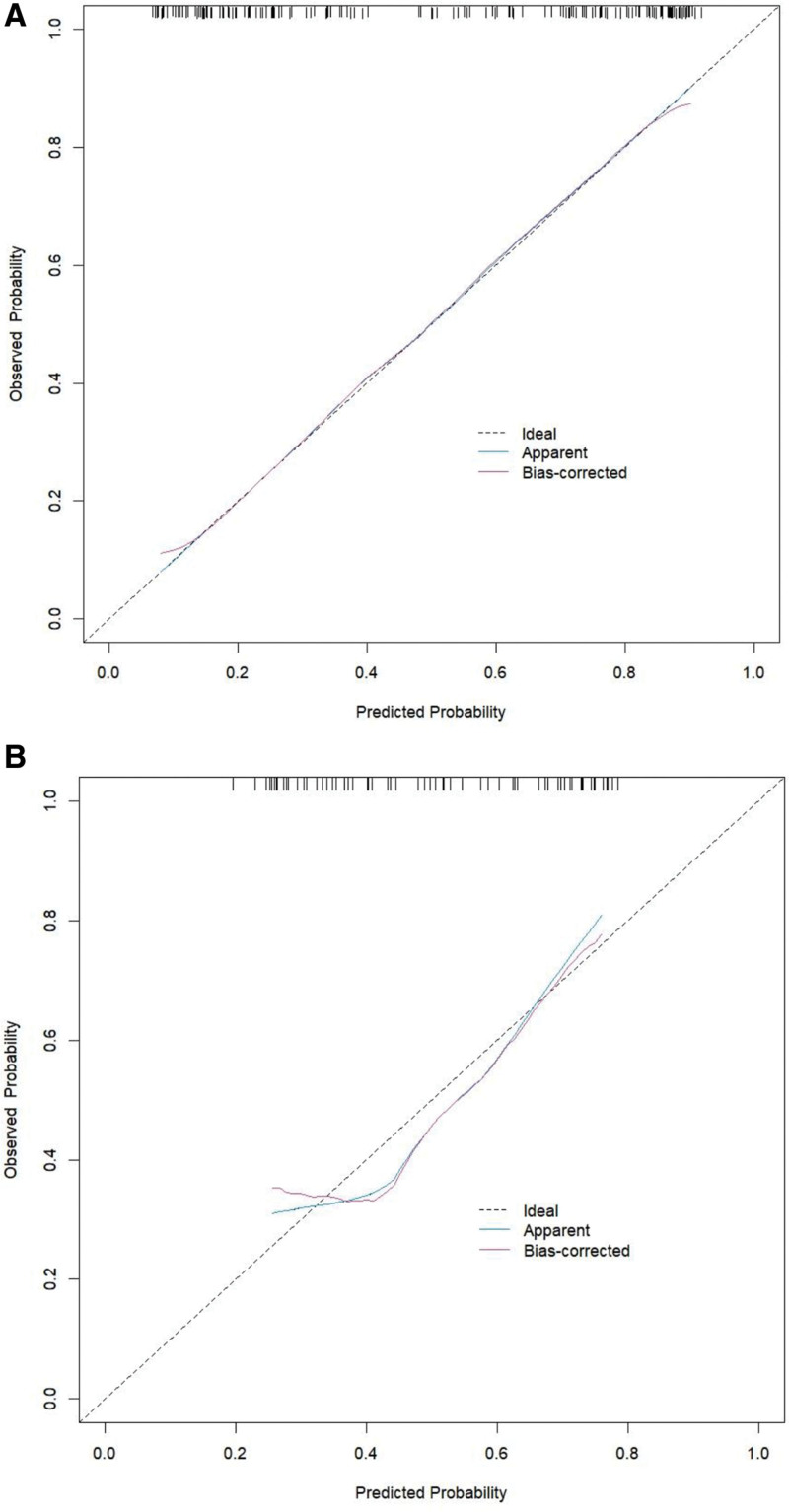
Calibration curve analysis for training set versus validation set nomogram models. (A) training set; (B) validation set.

The nomogram model demonstrated an AUC of 0.838 (95% CI: 0.775–0.902) in the training set and 0.712 (95% CI: 0.581–0.843) in the validation set, indicating good discriminatory ability and robust internal validation performance (Fig. 3). The calibration curve showed strong agreement between the predicted probabilities and the actual observed incidence. Hosmer–Lemeshow goodness-of-fit tests yielded *P* values of .865 and .558 for the training and validation sets, respectively (both > .05), suggesting excellent calibration of the model (Fig. 4).

**Figure 5.**
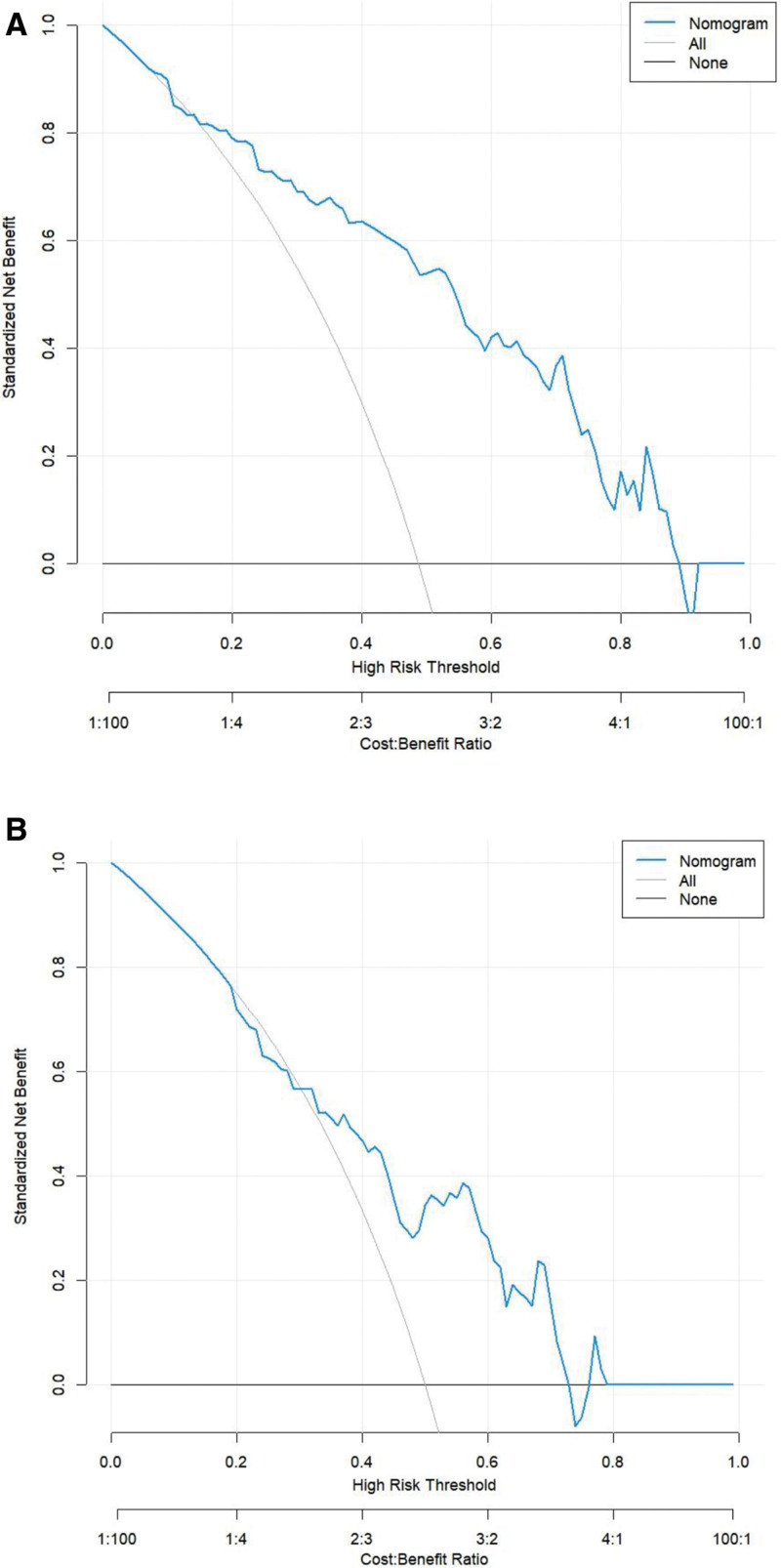
Decision curve analysis of training set versus validation set nomogram models. (A) training set; (B) validation set.

Decision curve analysis revealed that within a threshold probability range of 30 to 70%, the nomogram model provided a higher net benefit than both the “treat all” and “treat none” strategies. This indicates that the model offers clinical utility and practical value for supporting decision-making (Fig. 5).

## 4. Discussion

Based on the LASSO regression method, this study successfully developed and validated a nomogram model incorporating age, hypertension, LVEF, CHD, and lymphocyte count to predict the risk of acute exacerbation in patients with CHF. The model demonstrated AUC values of 0.838 and 0.712 in the training and validation sets, respectively, indicating good discriminatory ability.^[16]^ It is worth noting that although the AUC in the validation set was lower than that in the training set a common occurrence in predictive modeling the value of 0.712 still represents acceptable discriminative performance, particularly for clinical prediction models.^[15]^

The study population had a median age of 56.00 years and a median LVEF of 62.00%, suggesting that the cohort likely consisted primarily of patients with heart failure with preserved ejection fraction.^[18]^ These characteristics align with previous studies on risk factors for acute exacerbation in HFpEF populations.^[19]^ The 5 predictors selected by LASSO regression are all closely linked to the pathophysiology of AHFE. Age is a well-established prognostic factor in heart failure, associated with reduced cardiac reserve and polypharmacy.^[20]^ Hypertension, a major etiology of heart failure, often precipitates acute decompensation by increasing cardiac afterload and promoting ventricular remodeling.^[21]^

LVEF, as a quantitative measure of systolic function, has been widely validated as inversely associated with exacerbation risk.^[22]^ Notably, in this study, LVEF remained a significant predictor even in the relatively high range (median 62%), suggesting that even subtle variations within the normal range may carry prognostic significance in HFpEF.^[23]^ CHD, representing ischemic cardiomyopathy, contributes directly to acute worsening through myocardial ischemic events.^[24]^

Lymphocyte count, an emerging inflammatory marker, has garnered increasing attention for its association with heart failure outcomes.^[25]^ Lymphopenia may reflect a chronic inflammatory state, consistent with immune activation and inflammatory pathways in heart failure progression.^[26]^ Studies have shown that lymphocyte count is inversely correlated with B-type natriuretic peptide levels and independently predicts hospitalization risk in heart failure patients.^[27]^

Compared to previous studies, the novelty of this work lies in its specific focus on developing a prediction model for acute exacerbation in an HFpEF population.^[28]^ Most existing models primarily target patients with heart failure with reduced ejection fraction, whereas HFpEF exhibits distinct pathophysiological and clinical profiles.^[29]^ The use of LASSO regression in this study effectively addressed multicollinearity among variables, enhancing model stability and generalizability.^[30]^

Model calibration was confirmed using the Hosmer–Lemeshow test (*P* > .05), indicating good agreement between predicted probabilities and observed outcomes.^[31]^ Decision curve analysis further demonstrated the clinical utility of the model within a threshold probability range of 30 to 70%, supporting its value in individualized treatment decision-making.^[32]^ For example, high-risk patients identified by the model may benefit from intensified follow-up, optimized medication regimens, or earlier intervention.^[33]^

This study has several limitations. Firstly, the relatively small sample size may have limited the stability of the model, particularly in the validation set. Secondly, the single-center design may introduce selection bias, reducing the generalizability of the results. Additionally, the model did not include certain emerging biomarkers such as soluble stimulation of tumorigenesis 2 protein or galectin-3, which could potentially improve predictive performance. Thirdly, we did not have standardized, prospectively collected data to classify heart failure phenotype (heart failure with reduced ejection fraction/heart failure with mildly reduced ejection fraction/HFpEF). Because echocardiographic variables were incompletely and inconsistently recorded across centers, post hoc subtype assignment would likely introduce misclassification bias; therefore, we did not report subtype frequencies. Future prospective work will include protocolized imaging to enable robust, subtype-specific analyses. Finally, the lack of an external validation cohort necessitates further evaluation of the model’s applicability in broader populations.

In conclusion, the nomogram developed in this study based on 5 readily available clinical variables performed well in terms of discrimination, calibration, and net clinical benefit. It provides a practical tool for individualized risk assessment of AHFE in patients with CHF.

## Author contributions

**Conceptualization:** Da Yang, Fachao Shi.

**Data curation:** Jian Ma.

**Formal analysis:** Shuting Zhai.

**Writing – original draft:** Da Yang.

**Writing – review & editing:** Da Yang, Fachao Shi.
